# Rapidly increasing end-of-life care needs: a timely warning

**DOI:** 10.1186/s12916-017-0897-2

**Published:** 2017-07-10

**Authors:** Geoffrey Mitchell

**Affiliations:** Faculty of Medicine,University of Queensland, Herston Road, Herston, 4006 Australia

**Keywords:** End-of-life care, Primary care, Healthcare planning, Healthcare integration

## Abstract

Current trends in population ageing show that, in the near future, while more people will live longer, more will also die at any one time. Health systems, as well as individual practitioners, are only just becoming aware of the extent of this problem. Health systems will have to rapidly change practice to manage the number of people dying in the coming years, many with complex multimorbid conditions. The changes involved should include a personal recognition by all health professionals of their role in caring for the dying, and healthcare education must include end-of-life care management as part of the core curriculum. Further, health systems must improve integration between primary care and specialist clinicians to ensure the burden is shared efficiently across the system. Finally, it should be recognised that end-of-life care is not terminal care, but should be anticipated months or sometimes years ahead through advance care planning for known future complications by the patient’s clinical team, as well as by patients and their main carers, to manage crises as they ariserather than react to them once they arise.

Please see related article: https://bmcmedicine.biomedcentral.com/articles/10.1186/s12916-017-0860-2.

## Background

The rapid increase in population aging is a worldwide issue [1], although it is more pronounced in the developed world, bringing about obvious challenges. Nevertheless, a tacitly acknowledged but often ignored issue is the subsequently increasing number of people who will die annually. Etkind et al.’s [2] paper quantifies the size of this increase in England and Wales until 2040, and forcefully argues that health planners need to act now to be prepared for the massive change. The analysis could only be reliably performed up to 2040. Importantly, figures presented in this article indicate that the death rate for people over 65 will actually fall between 2014 and 2040, yet the number of deaths will rapidly increase. Thus, while relatively more people will survive to 2040, every one of these extra survivors, along with the increasing numbers who reach old age after 2040 will die at some stage after 2040, contributing to an increasing prevalence of deaths that will be seen after that cut-off date. The scale and rate of expansion of the problem noted by Etkind et al. [2] will continue to escalate well beyond 2040. This is a call all countries must heed.

Etkind et al. [2] estimate that the proportion of people approaching the end of life who could benefit from palliative care will rise to between 75% and 87.6% in 2040, with the greatest increases being in deaths from cancer and dementia. Most deaths will occur in people aged over 85. Thus, the authors call for an immediate, rapid escalation in the number of health professionals training in palliative care and geriatrics. They rightly point out that, while the number of specialist palliative care professionals needs to increase rapidly, many, if not most people who will die will be cared for by non-specialist professionals, such as in primary care, geriatrics or organ-based disciplines, with involvement of all health professionals in these settings. The burden of care has to fall on community-based professionals since there will simply not be enough inpatient beds and hospital-based staff to manage.

## Future policy imperatives

In addition to the call for an urgent rise in relevant specialist physician and nurse trainees, there are four further points of imperative immediate concern to health planners.

Firstly, end-of-life care is not a specialty, it is everyone’s business [3]. Thus, substantial training at all levels of the health system must be provided to achieve a strong foundation of knowledge and confidence in effective strategies to manage the challenges of maintaining quality of life as death approaches. Such training should be provided at undergraduate, vocational training and postgraduate levels. Furthermore, explicit management of end-of-life issues should be provided by all professionals. Indeed, the need will be so large that perhaps compulsory end-of-life training should be considered, much as compulsory CPR re-accreditation is now.

Second, natural ageing and comorbidities complicate the medical care of people at the end of their lives [4]. Etkind et al.’s [2] analysis focuses only on the disease leading to the reported death; it did not speculate on the added burdens of multimorbidity and frailty, which can impact on the patient, immediate family and the health system for years before the actual death [5]. Escalation of the burden of multimorbidity is now evident, and will increase exponentially. It is a facet of end-of-life care hidden in plain sight. Thus, research into multimorbidity and its management is a matter of urgency.

Third, end-of-life care is not terminal care. Preparation for end-of-life care should commence when an increasing burden of illness starts to incapacitate the sufferer – often several months or even years prior to death. Advance care planning to articulate the patient’s wishes at the end of life, clinical care planning to anticipate and minimise future health crises, and deliberate empowerment of patients and carers to self-manage these crises, all reduce the burden on the specialist sector [6]. These management strategies can be gradually introduced as the illness progresses. While advance care planning has received considerable attention, far less thought has been given to clinical care planning and patient preparation for crises.

Finally, health systems must acknowledge that business as usual will not cope with the oncoming pressure. Community-based care, particularly primary care and residential aged care, has much to offer people in the final months of life. Integration between traditionally siloed secondary and tertiary care and primary care, often with simple measures, yields major reductions on the demands on hospitals [7], with consequent cost savings [8]. However, implementing these measures requires changes in the working of these institutions, such that time and administrative support should be made available to facilitate joint care planning for patients already struggling.

## Conclusion

Modern healthcare has delivered spectacular improvements in health and longevity. Many feared diseases, which would have killed in midlife, have now been tamed. However, society remains reluctant to accept that this only delays the inevitable. Health systems must now invest as much effort on quality, integrated end-of-life care as they do into maintaining the population alive and free from prominent, alarming diseases.
